# Analysis of Grip Strength Thresholds for Stroke Management and Prevention in South Korean Older Adults

**DOI:** 10.3390/healthcare13070781

**Published:** 2025-03-31

**Authors:** Jong Hyeon Lee

**Affiliations:** Department of Sport Industry Studies, Yonsei University, Seoul 03722, Republic of Korea; leejh01@yonsei.ac.kr

**Keywords:** stroke, absolute grip strength, relative grip strength, older adults, KNHNES

## Abstract

Background/Objectives: Muscle weakness in older adults is associated with cardiovascular disease and all-cause mortality. However, its association with stroke prevalence remains underexplored. This study aimed to analyze the absolute grip strength (AGS) and weight-adjusted relative grip strength (RGS) thresholds for stroke prediction in South Korean older adults and to assess their sex-specific predictive ability. Methods: Data from 5185 older adults (2231 men; 2954 women) from the Korea National Health and Nutrition Examination Survey (KNHNES, 2014–2018) were analyzed using complex sampling methods. Receiver operating characteristic (ROC) curve analysis was performed to determine AGS and RGS thresholds and predictive performance, while multivariate logistic regression was used to adjust for confounders and to assess independent effects. Results: In older men, both the AGS and RGS demonstrated significant predictive ability for stroke, with AUCs of 0.637 and 0.623, respectively. In women, the AGS (AUC: 0.608) and RGS (AUC: 0.615) were predictive; however, only the RGS was significant for stroke management (odds ratio (OR): 3.026; 95% confidence interval (CI), 1.541–5.943). In men, AGS (OR: 3.544, 95% CI, 2.094–5.998) and RGS (OR: 2.585, 95% CI, 1.529–4.369) were significant. The stroke prediction thresholds were AGS 28.55 kg and RGS 0.47 for men and RGS 0.36 for women. Conclusions: The AGS and RGS provide practical indicators for stroke risk prediction based on sex-specific differences, highlighting their potential for public health and clinical applications. Future studies should investigate the stroke type, severity, and additional functional fitness indices.

## 1. Introduction

Stroke is a neurological injury caused by the sudden disruption of blood flow to the brain, resulting in symptoms such as muscle weakness, speech and vision impairment, and loss of coordination, emphasizing the need for early diagnosis and prevention [1,2]. Globally, approximately 12.2 million cases of stroke occur annually, and one in four people over the age of 25 years experiences a stroke during their lifetime. Stroke is recognized as a serious public health problem and is the second leading cause of death worldwide [3]. It is also a major cause of death in Korea, with over 600,000 cases occurring annually [4], and the mortality rate within one year after stroke is reported to be 20.1% as of 2022, at 18.5% in men and 22.1% in women [5]. As of 2022, the number of stroke cases in South Korea was reported to be 110,574 (61,988 men and 48,586 women). Also, the prevalence of stroke among Korean older adults is approximately 7.5%, which is more than four times higher than that observed in younger adults [5].

Mobility has been emphasized in rehabilitation sites for recovery from gait disturbances caused by stroke symptoms. However, muscle weakness due to sarcopenia and decreased neuromuscular control ability in older adults leads to reduced physical activity. This decline, combined with changes in blood flow dynamics, impairs venous return, slows the recovery rate during rest, and increases the risk of stroke due to cerebrovascular dysfunction caused by thrombosis [6]. Muscles, as key metabolic organs, can weaken due to muscle loss, disrupting blood sugar and lipid metabolism, and increasing stroke risk. Stroke-induced upper motor neuron damage exacerbates muscle dysfunction, leading to reduced muscle fiber size, motor unit loss, altered recruitment rates, and reduced walking speed and endurance [7]. Therefore, the need for an exercise program that not only improves muscle mass and strength but also complements traditional function-oriented interventions has been emphasized, as evidence suggests that stroke recovery is facilitated by enhancing muscle strength [8]. Therefore, identifying the muscle strength level through one-repetition maximum (1RM) measurement before starting strength training is generally emphasized; however, in the case of neurological damage due to stroke, muscle endurance at the submaximal level is more closely related to functional recovery in daily life than one-time maximal muscle strength [9,10,11,12]. Consequently, exercise intensity of an effective muscle strength improvement program is 70% of the maximal muscle strength [13], and 1RM measurement can be dangerous and unnecessary waste in the rehabilitation of stroke patients [8]. Grip strength (GS) is easy to measure, reflects overall muscle strength in older adults, and effectively predicts stroke risk, making it valuable for stroke prevention and rehabilitation [14]. Large-scale cohort studies, such as the China Health and Retirement Longitudinal Study (CHARLS), have demonstrated that changes in GS over time are associated with stroke incidence, reinforcing its role in stroke prevention [15]. Additionally, while the Jamar dynamometer is a standard tool for GS assessment, evidence suggests that electronic dynamometers provide comparable accuracy at a lower cost, making them a practical alternative for large-scale applications [16]. The importance of measurement is further highlighted, as it has been revealed that when GS is weak, essential daily movements such as getting up from a chair, walking, and climbing stairs are critically limited [17,18]. Stronger GS in stroke patients is linked to earlier hospital discharge [19,20] and better daily function, showing high correlations with the Frenchay Arm Test (r = 0.91), Motor Club Assessment (r = 0.86), and Peg Test (r = 0.79). Grip strength training also enhances cognitive function by improving the efficiency of the white matter network in stroke patients [21]. These findings suggest that GS may be an effective method for the prevention and treatment of stroke.

Although GS is most widely associated with all-cause mortality [22,23,24,25], several thresholds of GS have been reported to predict diseases, with values of 28 kg for men and 18 kg for women suggested as the criteria for determining sarcopenia in Asians [26]. For diabetes, the threshold is 28.3 kg for men and 23.4 kg for women, regardless of race [14]. Similar figures have been reported for older adults; to safely lift an object weighing more than 10 kg without injury, a grip strength of more than 28.5 kg for men and 18.5 kg for women is required [27].

Thus, the core mechanisms of post-stroke recovery and the effects of muscle strength intervention, as assessed by GS, have been presented [17,28]; however, the muscle strength threshold for early and specific prevention and management of stroke has not been investigated to date. In addition, muscle strength has been suggested to be related to cardiovascular mortality rather than disease prevention; therefore, physical fitness values, including muscle strength, that prevent disease occurrence itself rather than death, should be suggested for early disease prediction to prevent the occurrence of fatal conditions. Therefore, deriving thresholds for both absolute grip strength (AGS) and relative grip strength (RGS) is essential to optimize tailored prevention and rehabilitation programs, accounting for sex differences in physique and muscle strength distribution among older adults. AGS is measured in kilograms using a dynamometer, and relative grip strength, RGS, is calculated by dividing AGS by body weight or body mass index (BMI). RGS is commonly used to minimize the confounding effect of body weight when examining the relationship between grip strength and disease prevalence [29].

While previous studies, such as Liu et al. (2021) [15], have established the link between low GS and increased stroke risk in the Chinese population, our study further distinguishes between AGS and RGS in predicting stroke risk among South Korean older adults. Additionally, we incorporate key metabolic risk factors such as hypertension, dyslipidemia, and diabetes mellitus to strengthen our predictive model. This study aims to identify AGS and RSG thresholds for stroke prevention and management using data from the Korea National Health and Nutrition Examination Survey (KNHNES) [30], a nationally representative dataset of older adults in South Korea, to evaluate the classification performance of AGS and RGS in stroke prediction, explore their clinical applicability, and assess the potential preventive impact of maintaining GS above the identified thresholds. Also, our research considers the longitudinal implications of grip strength changes and their potential interactions with metabolic risk factors, which may further refine stroke prediction models. These findings will provide evidence to guide public health policies that address sex differences in stroke risk and support programs aimed at preventing muscle strength decline in older adults.

## 2. Materials and Methods

### 2.1. Data Acquisition and Participants

This study used data from the KNHNES, conducted by the Korea Centers for Disease Control and Prevention. Raw data from 2014 to 2018, with GS data, were used after submitting the plan and purpose of this study and receiving approval. In this study, older adults were defined as those aged 65 years or older, and the age group was set as the subpopulation. In this study, 5185 participants (2231 men and 2954 women) with both stroke and GS indicators were analyzed.

### 2.2. Research Variables

#### 2.2.1. Stroke Prevalence

In this study, to identify the prevalence of stroke in older adults, a binary variable was reconstructed for participants who had been diagnosed with stroke, currently had stroke, were receiving stroke treatment, or were experiencing stroke–stroke sequelae. If none of the above applied, the participants were classified as having no stroke.

#### 2.2.2. Grip Strength

In this study, GS (kg) data were collected from the KNHNES (2014–2018), with measurements performed thrice for each hand (left and right) using a digital grip dynamometer (TTK 5401, Takei, Japan) following standardized procedures. Participants were instructed to sit upright with their elbows flexed at 90 degrees, their forearms in a neutral position, and their wrists slightly extended (0–30 degrees). They were asked to squeeze the dynamometer with maximum effort for a few seconds without any additional body movement. Trained examiners conducted all assessments to ensure measurement, accuracy, and consistency. Individuals unable to undergo GS measurement due to conditions such as arm, hand, or thumb loss or fracture; hand paralysis; use of a cast or bandage on the hand or wrist; history of wrist surgery or arthritis within the past three months; or recent hand pain, stiffness, or worsening symptoms were excluded from the measurements. The highest value among the six measurements was considered as the AGS. Additionally, the RGS was calculated as the AGS divided by body weight.

#### 2.2.3. Covariates

Age, household income, marital status, and educational level were adjusted to analyze the relationship between GS and stroke in older adults. In addition, health behavioral variables such as alcohol consumption, smoking, obesity, and strength training practice were adjusted. Also, our analysis controlled metabolic conditions such as physician-diagnosed hypertension, dyslipidemia, and diabetes mellitus, as these factors have been previously identified as significant contributors to stroke risk [31]. While heart failure and atrial fibrillation [32] were not included due to dataset limitations, we acknowledge their potential relevance and suggest future research explore their role in grip strength-related stroke prediction. Household income was classified into four quartiles. Education level was reclassified as “elementary school” for Seodang/Chinese schools, no school, and elementary schools; middle school and high school items were used as they were; and for 2/3-year college, 4-year college, and graduate school or higher, a variable item was reclassified as “college or higher”. Alcohol consumption was measured using annual drinking frequency data, and variables were constructed with the items “Never drank in the past year”, “Once a week or less”, “2–3 times a week”, and “4 or more times a week”. Smoking data were reconstructed as binary variables in which data on current smoking status were surveyed, with items such as currently smoking or occasionally smoking as “smoking”, in the past but currently quitting, and never smoking as “non-smoking”. Obesity was assessed using body mass index (BMI) data, and pregnant women were excluded from the analysis. BMI classifications were as follows: underweight (less than 18.5 kg/m^2^), normal weight (18.5–24.9 kg/m^2^), and obese (25 kg/m^2^ or higher). Strength training practice was categorized as a binary variable: individuals who did not engage in strength training were classified as “no practice”, while those who performed strength training at least once per week were classified as “practice”.

### 2.3. Data Analysis

To determine the AGS and RGS cutoff values for stroke prevention, sex-stratified receiver operating characteristic (ROC) curve analysis was conducted, with the area under the curve (AUC) evaluated under a null hypothesis value of 0.5. The highest Youden index was used to define the cutoff thresholds [33], and the stroke prediction accuracy was assessed using a confusion matrix to calculate sensitivity, specificity, positive predictive value (PPV), negative predictive value (NPV), and accuracy [34]. A complex sample analysis method was used to represent the older South Koreans. Variance estimation strata (kstrata) and cluster variables (psu) were set, and integrated weights were calculated based on the survey sample size for each year (2014: wt_ivex × 7550/39,199; 2015: wt_ivex × 7380/39,199; 2016: wt_ivex × 8150/39,199; 2017: wt_ivex × 8127/39,199; 2018: wt_ivex × 7992/39,199). Participants aged 65 years or older were analyzed as a subpopulation, with sex-specific filters created to minimize bias in population estimation. Data analyses reflected the estimated population size using the finite-population correction method. Descriptive statistics (mean and standard error (SE)) and cross-tabulation were performed to analyze the demographic and GS distributions by sex, with independence tests for categorical variable differences. Stroke risk was analyzed by dividing the participants into weak and strong GS groups based on the thresholds. Adjusted odds ratios (aOR) and 95% confidence intervals (CIs) were derived using a complex sample logistic regression model with four adjustment levels: Model 1 (age, income, and education), Model 2 (Model 1 + alcohol consumption, smoking, hypertension, diabetes, and dyslipidemia), and Model 3 (Model 2 + strength training practice). All analyses were performed using SPSS ver. 27.0, with statistical significance set at *p* < 0.05.

### 2.4. Ethics Statement

This study utilized KNHNES open-source, anonymized personal data. Since the KNHNES is conducted directly by the state for public welfare in accordance with Article 2, Paragraph 1, of the Bioethics Act and Article 2, Paragraph 2, Subparagraph 1, of the Enforcement Decree of the same Act, it can be conducted without review by the Research Ethics Review Committee. Nevertheless, we disclose that the data for the 6th period, 2014 and 2015, were collected after review (Approval Number: 2013-12EXP-03-5C). In addition, the data for the 7th period (2016, 2017, 2018) were collected without review, according to the opinion of the Research Ethics Review Committee of the Korea Disease Control and Prevention Agency. Informed consent was obtained from all participants at the time of data collection, and all data collection and analysis procedures were conducted in compliance with the research ethics guidelines of the Declaration of Helsinki.

## 3. Results

### 3.1. ROC Curve Analysis of Grip Strength for Stroke Management

The results of the ROC curve analysis were used to determine the AGS and RGS thresholds for stroke prevention, and are shown in Table 1. For older men, the AGS had an AUC of 0.637 (95% CI, 0.591–0.684) and an accuracy of 0.74, while the RGS had an AUC of 0.623 (95% CI, 0.574–0.672) and an accuracy of 0.65. For older women, the AGS had an AUC of 0.608 (95% CI, 0.557–0.659) and an accuracy of 0.69, while the RGS had an AUC of 0.615 (95% CI, 0.556–0.664) and an accuracy of 0.49. Detailed results are presented in Table 1.

### 3.2. Participant Characteristics

Descriptive statistics for age, AGS, and RGS values of older adults and the distribution according to the calculated GS thresholds are shown in Table 2.

The results for the prevalence of stroke and other adjusted variables are shown in Table 3.

The diagnosis rate for stroke tended to be significantly higher in men than in women (x^2^ = 9.544, *p* = 0.008). In addition, there were significant differences by sex in household income (x^2^ = 80.611, *p* = 0.000), education level (x^2^ = 654.672, *p* < 0.000), hypertension (x^2^ = 9.967, *p* = 0.008), dyslipidemia diagnosis (x^2^ = 129.587, *p* = 0.000), alcohol consumption (x^2^ = 415.341, *p* < 0.000), smoking (x^2^ = 407.538, *p* = 0.000), and strength training practice (x^2^ = 305.324, *p* = 0.000).

### 3.3. Difference in Stroke Prevalence by Grip Strength Level

In Model 3, which included all adjusted variables, older men with AGS below the threshold showed a significantly higher stroke prevalence risk (354.4%, 95% CI: 2.094–5.998, *p* = 0.000), and those with RGS below the threshold also showed a significantly higher risk (258.5%; 95% CI 1.529–4.369; *p* = 0.000) compared to those above the threshold. Similarly, for older women, those with RGS below the threshold had a significantly higher stroke prevalence risk (302.6%; 95% CI 1.541–5.943; *p* = 0.001). However, the association between the AGS and stroke prevalence in older women was not statistically significant after adjustment in Model 3. The detailed results are presented in Table 4.

## 4. Discussion

This study is the first to present the AGS and RGS thresholds for stroke prediction in older adults in South Korea, using large-scale open-source data, with sex-specific differentiation. By analyzing the KNHANES data, we identified grip strength thresholds for stroke risk and offer a reference for strength-training goals in stroke prevention and management.

The AUC of the older adults’ GS threshold derived in this study was 0.608–0.637, which is generally considered fair, and is similar to those previously reported for cardiovascular disease and mortality (AUC: 0.65–0.75) [35], metabolic syndrome (AUC: 0.65–0.71) [36], frailty (AUC: 0.6–0.7) [14], and all-cause mortality (AUC: 0.66–0.72) [35]. However, these studies generally considered multiple diseases in an integrated manner and were not predictive studies for any single specific disease. Stroke is a major cause of death worldwide, with fatal sequelae, and is a serious public health concern, especially in older age groups [37]. Therefore, the classification performance evaluation results of this study are significant for clinical evaluation and establishment of prevention strategies for stroke risk and its clinical significance. In addition, compared to Liu et al. (2021) [15], who reported an HR of 1.89 for weak GS in predicting stroke, our study found similar trends, though our AUC values indicate only fair discrimination. While AGS and RGS provide additional insights into stroke risk, their predictive performance should be interpreted in the context of established stroke risk models such as the Framingham Stroke Risk Score [38]. Combining grip strength with these models may enhance their predictive accuracy and clinical applicability.

Among the derived GS thresholds, the AGS for men was 28.55 kg and for women was 17.2 kg, which is similar to the AGS thresholds for cardiovascular and metabolic diseases for men (26–30 kg) and for women (16–20 kg) [35], and the thresholds for all-cause mortality of 26–28 kg for men and 15–18 kg for women [39]. In particular, RGS assessment is needed to supplement the risk that is difficult to capture with the AGS alone in older adults with high body weight. The threshold related to cardiovascular disease and all-cause mortality was 0.45–0.55 for men and 0.35–0.45 for women [35], and the Asian Working Group for Sarcopenia (AWGS) suggested a threshold of 0.40 for men and 0.30 for women in the diagnosis of sarcopenia [26]. This study suggests that the management of RGS may be more essential for managing stroke, as higher values of 0.47 for men and 0.36 for women were presented.

The sensitivity of AGS for men was 75.9%, demonstrating a strong performance in identifying stroke patients, whereas the RGS exhibited higher specificity (56.5%) than the AGS, indicating its usefulness in identifying individuals without stroke risk. Among older women, the AGS sensitivity was 70.4% and the RGS specificity was higher at 72.1%, suggesting better predictive accuracy for the AGS in identifying stroke risk. Although the PPV for all GS measures was below 12%, limiting their accuracy in predicting actual stroke cases, the NPV exceeded 95%, indicating a high predictive performance for the non-occurrence of strokes. This suggests that grip strength testing could serve as a key determinant in identifying individuals at low stroke risk, potentially reducing the need for additional assessments. Therefore, the AGS is considered a suitable early screening tool for stroke because of its high sensitivity and overall accuracy, whereas the RGS, with its relatively high specificity, serves as a complementary measure. Therefore, evaluating both the AGS and RGS is recommended to enhance the prediction of stroke risk in older adults.

In addition, this study attempted to increase the explanatory power of the results by adding the results of the multivariate logistic regression analysis adjusted for sociodemographic factors, health behaviors, comorbidities, and strength training practice to the results of the ROC curve analysis. The analysis results showed that the AGS in men had a relatively superior predictive performance to the RGS, with a higher AUC, and its OR was also higher than that of the RGS (3.544 vs. 2.585). This suggests that although RGS is important in stroke management, AGS is a relatively more powerful variable and was statistically significant in the fully adjusted Model 3. In contrast, in women, RSG showed a higher predictive performance relative to body weight. In Model 3, AGS did not statistically significantly predict stroke, whereas RGS was identified as a significant variable, revealing the possibility that assessing muscle strength relative to body weight may be more useful for predicting stroke in older women. These results suggest that GS weakness evaluated in a multivariate model should be utilized as a useful risk factor, although it does not have high predictive performance as a single variable for stroke prediction. However, the wide confidence intervals observed for RGS suggest potential heterogeneity within our dataset, which may be influenced by variations in muscle mass distribution, lifestyle factors, and unmeasured confounders. Future studies should aim to include larger sample sizes and more diverse populations to enhance the robustness of grip strength-based stroke prediction models.

This study highlights the need to consider sex-specific utilization of grip strength indicators in older adults. Among older men, higher muscle mass relative to body weight supports the predictive utility of both the AGS and RGS for stroke risk, with the AGS showing a stronger predictive power. This may be attributed to the closer association between absolute muscle mass and the risks of stroke, cardiovascular diseases, and all-cause mortality in men; therefore, weakened muscle contraction and relaxation due to sarcopenia may exacerbate endothelial dysfunction, increase vascular inflammation and stress, and impair peripheral circulation, particularly in the brain, making older men more vulnerable to these mechanisms [40,41,42,43]. In contrast, older women generally exhibit lower muscle mass and higher body fat, with RGS identified as a significant predictor of chronic conditions such as diabetes and stroke [44,45,46]. This study also found that RGS was a relevant variable for predicting stroke in older women. Notably, the rate of strength training participation among older women was significantly lower than that among men, suggesting a greater reliance on aerobic activities such as walking rather than high-intensity or resistance exercises [47]. To improve stroke prevention in women, interventions should focus on increasing lean mass through strength training, while avoiding excessive restrictions on carbohydrates and proteins that may impair muscle maintenance. As maximal strength gains from resistance training show no sex differences [48], future research should explore social factors, body image perceptions, and attitudes toward exercise that influence strength training participation to inform public health policies.

A rapid decrease in vascular elasticity is one of the representative mechanisms of aging, which causes poor blood flow to the brain, and the resulting decrease in functional physical strength leads to a rapid decrease in muscle strength, thus increasing the risk of stroke [49]. Therefore, regular strength training can be a preventive measure, because it lowers systemic inflammation and improves blood circulation [50]. Lower extremity strength plays a major role in improving cerebral blood flow and maintaining peripheral vascular function. Because the vicious cycle of sarcopenia resulting from stroke is likely to be repeated [50,51], the importance of maintaining muscle strength and checking GS is further emphasized [52]. Therefore, evaluating the effectiveness of a strength improvement program by considering the AGS and RGS thresholds of older men and the RGS of older women may be useful in stroke prevention. However, while GS is a valuable biomarker for stroke risk, it should be considered in conjunction with metabolic conditions such as hypertension, dyslipidemia, and diabetes mellitus. Its role as a functional indicator complements traditional cardiovascular risk assessments and may enhance risk stratification in clinical and public health settings.

This study had several limitations. First, because of its cross-sectional design using large open-source data, causality between grip strength and stroke could not be established. Our study does not account for longitudinal changes in grip strength, which may play a crucial role in stroke risk prediction. Future research should incorporate longitudinal data to evaluate how changes in grip strength over time influence stroke incidence and severity. Additionally, it did not differentiate between ischemic stroke-induced hemiparesis, which can cause severe asymmetry in grip strength, and hemorrhagic stroke and its functional deficits [53,54]. This relationship may vary according to stroke phase, and future studies should explore nonlinear associations. Also, future research should differentiate between ischemic and hemorrhagic stroke subtypes, as these conditions may have distinct pathophysiological mechanisms that influence the role of muscle strength in stroke risk. Second, the low positive predictive value of the AGS and RGS stroke prediction cut-offs warrants caution in their interpretation. For better stroke management and identification, specialized stroke diagnostic tools such as the Cincinnati prehospital stroke scale (CPSS) [55] and LA Prehospital Stroke Scale [56] may be used. Third, this study did not include other muscle-strength-related measures. Although GS is a common proxy, future research should consider functional fitness measures such as calf circumference, and the strength, assistance with walking, rising from a chair, climbing stairs, and falls (SARC-F) questionnaire to identify sarcopenia-related patient outcomes [57]; adding these tests’ thresholds and their association with stroke risk could further enhance predictive accuracy. Fourth, handedness and inter-hand strength differences were not specifically analyzed in this study. Since the highest GS value among both hands was used as AGS, potential effects of hand dominance and stroke-induced asymmetry were not accounted for. However, a previous study [15] has also adopted the approach of selecting the highest value from the dominant hand only, whereas our study followed the Hong et al. (2021) [58] method, which considers the maximum value among six trials from both hands to ensure a more comprehensive assessment of grip strength. Future studies should investigate how handedness and stroke laterality influence GS-based stroke risk prediction. In addition, future studies could apply propensity score matching to address the imbalance between patients with stroke and controls. Although this study aimed to provide a practical and intuitive indicator for stroke risk prediction based on GS thresholds, without additional statistical adjustments, its significance remains unclear. Finally, the data were limited to South Korea; therefore, applying the findings to the global older adult populations may be challenging. Future cross-national and racial comparative studies are required.

## 5. Conclusions

The significant cut-off values for stroke risk management in older adults in South Korea were determined as an AGS of 28.55 kg and RGS of 0.47 for men, and an RGS of 0.36 for women. Based on these thresholds, a multivariate model adjusted for demographic variables, comorbidities, health behaviors, and strength training showed significant prediction of stroke risk, excluding AGS in women. However, because of the relatively low AUC and PPV, these grip strength thresholds should be used primarily as tools for early prevention rather than for definitive stroke diagnosis in community health centers, hospitals, and nursing facilities. Regular measurement of both AGS and RGS can help identify high-risk older adults with values below the threshold, allowing for early intervention and preventive measures. Future studies should explore the integration of GS into established stroke risk models and investigate its predictive utility in different population subgroups. Additionally, examining the interaction between grip strength and other functional fitness measures may provide a more comprehensive understanding of its role in stroke prevention and management.

This study confirmed that AGS in older men and RGS in older women are more strongly associated with stroke risk. To maximize stroke prevention, older men should focus on strength-building exercises, while women should benefit from weight management, and strength training, within a comprehensive program.

## Figures and Tables

**Table 1:** ROC curve analysis of absolute grip strength and relative grip strength for stroke prediction.

		AUC	95% CI	Cut off (kg)	Sensitivity	Specificity	PPV	NPV	Accuracy
Men	AGS	0.637	0.591–0.684	28.55 ***	0.759	0.469	0.12	0.95	0.74
	RGS	0.623	0.574–0.672	0.47 ***	0.658	0.565	0.10	0.95	0.65
Women	AGS	0.608	0.557–0.659	17.25 ***	0.704	0.493	0.08	0.96	0.69
	RGS	0.615	0.556–0.664	0.36 ***	0.481	0.721	0.07	0.97	0.49

AUC: area under ROC curve; CI: confidence interval; PPV: positive predictive value; NPV: negative predictive value; AGS: absolute grip strength; RGS: relative grip strength. *** *p* < 0.001.

**Table 2:** Descriptive analysis and frequency distribution of participants’ age and grip strength.

Men			Women		
Age (year)	72.33 ± 0.128		Age (year)	73.38 ± 0.116	
AGS (kg)	33.071 ± 0.185		AGS (kg)	19.503 ± 0.134	
RGS (kg)	0.512 ± 0.003		RGS (kg)	0.350 ± 0.002	
	n	B		n	B
AGS < 28.55 kg	550	473,807	AGS < 17.25 kg	824	789,010
AGS ≥ 28.55 kg	1589	1,446,953	AGS ≥ 17.25 kg	1873	1,686,428
RGS < 0.47 kg	764	654,058	RGS < 0.36 kg	1425	1,316,331
RGS ≥ 0.47 kg	1374	1,265,504	RGS ≥ 0.36 kg	1270	1,157,139

AGS: absolute grip strength; RGS: relative grip strength; n: unweighted frequency; B: estimated population size.

**Table 3:** Participant characteristics by stroke prevalence and adjusted variables.

Variables	Men			Women				
	n	B	%	n	B	%	x^2^	*p*
No stroke	2076	1,863,169	42.0	2804	2,574,921	58.0	9.544	0.008
Stroke	155	143,747	50.9	150	138,518	49.1		
Household income: 1Q	261	243,127	51.0	246	233,971	49.0	80.611	0.000
2Q	374	348,722	48.7	386	366,786	51.3		
3Q	663	590,363	47.1	745	661,834	52.9		
4Q	915	801,723	36.1	1558	1,416,140	66.5		
Married	2212	19,920,213	42.5	2935	2,697,069	57.5	0.374	0.568
Not married	19	14,903	47.7	19	16,369	52.3		
Elementary school	683	595,548	25.8	1872	1,709,850	76.4	654.672	0.000
Middle school	451	402,845	51.7	425	375,786	48.3		
High school	546	479,857	61.6	316	299,020	38.4		
College or higher	406	383,932	71.7	153	151,233	28.3		
No hypertension	1034	934,806	44.9	1218	1,146,452	55.1	9.967	0.008
Hypertension	1182	1,058,571	40.5	1717	1,552,700	59.5		
No dyslipidemia	1686	1,520,699	48.1	1764	1,638,152	51.9	139.587	0.000
Dyslipidemia	529	472,019	30.8	1171	1,061,000	69.2		
No diabetes	1707	1,553,779	42.6	2290	2,095,785	57.4	0.026	0.897
Diabetes	508	438,939	42.3	643	598,528	57.7		
No alcohol consumption	548	478,407	42.9	683	637,452	57.1	415.341	0.000
Once a week or less	763	682,570	44.9	948	836,444	55.1		
2–3 times a week	337	412,395	77.3	91	91,798	22.7		
4 times a week or more	359	336,924	88.7	55	55,529	11.3		
No smoking	1787	1,596,397	38.3	2810	2,570,487	61.7	407.538	0.000
Smoking	400	367,397	86.9	63	55,600	13.1		
Normal weight	1044	930,407	45.9	1189	1,094,519	54.1	4.759	0.168
Underweight	70	64,815	46.7	72	73,994	53.3		
Obese	659	607,614	42.5	910	822,744	57.5		
No strength training	1484	1,311,133	36.4	2503	2,289,759	63.6	305.324	0.000
Strength training practice	604	554,118	68.4	274	256,368	31.6		

n: unweighted frequency; B: estimated population size; %: rates by stroke prevalence; Q: quartile.

**Table 4:** Analysis of the relationship between older adults’ handgrip strength and stroke prevalence.

Sex	Model		OR	95% CI	*p*
Men	Unadjusted	AGS (kg)	3.642 ***	2.471–5.367	0.000
		RGS (kg)	2.624 ***	1.770–3.889	0.000
Women		AGS (kg)	1.831 **	1.202–2.790	0.005
		RGS (kg)	2.336 ***	1.478–3.693	0.000
Men	Model 1	AGS (kg)	3.431 ***	2.168–5.428	0.000
		RGS (kg)	2.241 ***	1.461–3.436	0.000
Women		AGS (kg)	1.767 *	1.139–2.740	0.011
		RGS (kg)	2.497 ***	1.523–4.092	0.000
Men	Model 2	AGS (kg)	3.600 ***	2.121–6.112	0.000
		RGS (kg)	2.627 ***	1.556–4.436	0.000
Women		AGS (kg)	1.972	0.926–4.197	0.078
		RGS (kg)	3.104 **	1.569–6.140	0.001
Men	Model 3	AGS (kg)	3.544 ***	2.094–5.998	0.000
		RGS (kg)	2.585 ***	1.529–4.369	0.000
Women		AGS (kg)	1.899	0.890–4.055	0.097
		RGS (kg)	3.026 **	1.541–5.943	0.001

OR, odds ratio; CI, confidence interval; Model 1: adjusted for age, household income, marital status, and educational level; Model 2: Model 1 + alcohol consumption, smoking, diagnosed hypertension, diabetes, and dyslipidemia; Model 3: Model 2 + strength training practice; AGS, absolute grip strength; RGS, relative grip strength * *p* < 0.05; ** *p* < 0.01; *** *p* < 0.001.

## Data Availability

The data supporting the findings of this study are available from the Korea National Health and Nutrition Examination Survey (KNHNES). For further details, please contact the corresponding author.

## Abbreviations

The following abbreviations are used in this manuscript:

1RM	One repetition maximum
GS	Grip strength
AGS	Absolute grip strength
RGS	Relative grip strength
ROC	Receiver operating characteristics
AUC	Area under the curve
OR	Odd ratio
CI	Confidence interval
CPSS	Cincinnati Pre-hospital Stroke Scale
